# The Potential Role of Etanercept in the Management of Post-stroke Pain: A Literature Review

**DOI:** 10.7759/cureus.36185

**Published:** 2023-03-15

**Authors:** Andrew M Joseph, Monica Karas, Cesar E Jara Silva, Melissa Leyva, Abdus Salam, Mehul Sinha, Yonathan Aliye Asfaw, Ayesha Fonseca, Steven Cordova, Marlon Reyes, Jonathan Quinonez, Samir Ruxmohan

**Affiliations:** 1 Osteopathic Medicine, Nova Southeastern University Dr. Kiran C. Patel College of Osteopathic Medicine, Davie, USA; 2 Osteopathic Medicine, Nova Southeastern University Dr. Kiran C. Patel College of Osteopathic Medicine, Fort Lauderdale, USA; 3 General Surgery, Khyber Medical College, Peshawar, PAK; 4 Research, International Society for Chronic Illnesses, Vadodara, IND; 5 Surgery, Kasturba Medical College, Mangalore, IND; 6 Internal Medicine, University of Gondar, Gondar, ETH; 7 College of Medicine, American University of Integrative Sciences, Bridgetown, BRB; 8 Neurology, St. Matthew's University School of Medicine, Miami, USA; 9 Allopathic Medicine, American University of Antigua, Osbourn, ATG; 10 Neurology/Osteopathic Neuromuscular Medicine, Larkin Community Hospital, Miami, USA; 11 Neurocritical Care, University of Texas Southwestern Medical Center, Dallas, USA

**Keywords:** stroke, tumor necrosis factor alpha, etanercept, pain management, post-stroke pain, tnf alpha, ischemic stroke

## Abstract

Strokes are the second leading cause of death and disability worldwide. The brain injury resulting from stroke produces a persistent neuroinflammatory response in the brain, resulting in a spectrum of neurologic dysfunction affecting stroke survivors chronically, also known as post-stroke pain. Excess production of tumor necrosis factor alpha (TNF alpha) in the cerebrospinal fluid (CSF) of stroke survivors has been implicated in post-stroke pain. Therefore, this literature review aims to assess and review the role of perispinal etanercept in the management of post-stroke pain.

Several studies have shown statistically significant evidence that etanercept, a TNF alpha inhibitor, can reduce symptoms present in post-stroke syndrome by targeting the excess TNF alpha produced in the CSF. Studies have also shown improvements in not only post-stroke pain but also in traumatic brain injury and dementia. Further research is needed to explore the effects of TNF alpha on stroke prognosis and determine the optimal frequency and duration of etanercept treatment for post-stroke pain.

## Introduction and background

Ischemic heart disease and cerebrovascular incidents are among the top causes of mortality worldwide [1]. Vascular diseases like hypertension and diabetes quickly become leading risk factors for the onset of cerebrovascular incidents [1]. While several studies have discussed the pathophysiology of strokes in patients with vascular disease, few have investigated the treatments for post-stroke pain that many patients experience [2]. The severity of post-stroke pain can be associated with the level of inflammation present, which can ultimately be debilitating and may lead to disability [2]. Tumor necrosis factor alpha (TNF alpha) plays a major role in causing and maintaining this inflammation in patients with vascular disease or post-stroke pain [1]. Etanercept, a TNF alpha inhibitor, can help reduce the inflammation caused by TNF alpha [2]. Therefore, this literature review aims to assess and review the role of etanercept in the management of post-stroke pain.

Post-stroke pain

A stroke is one of the leading causes of global morbidity and mortality [3]. The annual incidence of strokes in the United States is approximately 795,000 [3]. For adults aged 25 years and older, the lifetime risk of stroke is about 25% [4]. Chronic pain presents in approximately 11-55% of patients after experiencing a stroke [5]. The functional instability from the lack of blood supply in the brain has acute and chronic consequences. The sequelae often seen in survivors include weakness, paralysis, numbness, and pain. Neuropathic pain or post-stroke pain syndrome presents a significant burden to patients. The prevalence of this disease ranges between 1% and 35% [6]. This can be attributed to the variabilities in the definition of pain category, inclusion criteria, and length of time of patient observation [7]. Post-stroke pain syndrome can appear six months to 10 years after the stroke episode [7].

The two major categories of strokes are ischemic and hemorrhagic, which can be further subdivided into different clinical courses and causes [8]. Approximately 80% of strokes are caused by ischemic cerebral infarctions, while 20% are due to hemorrhage [8]. Some causes of ischemic stroke include thrombosis in large or small vessels, systemic hypoperfusion, and embolism. Large vessel thrombosis commonly occurs extracranially, usually in the common and internal carotids, and intracranially in the circle of Willis [8]. These vessels' common pathologies include atherosclerosis, dissection, fibromuscular dysplasia, and arteritis [8]. Causes of hemorrhagic stroke include intracerebral hemorrhage and subarachnoid hemorrhage, which results in an increase in the intracranial pressure limiting the proper hemodynamic circulation [8].

Despite the revascularization of brain tissue from a stroke, significant consequences affect the patient’s quality of life [5]. Chronic pain represents one of the major burdens following a stroke [5]. Manifestations of chronic pain include central post-stroke pain, musculoskeletal pain, shoulder pain, painful spasticity, and tension-type headache [5]. Furthermore, the clinical presentation is associated with central and peripheral neuropathic pain syndromes, which occur spontaneously 85% of the time [9]. On average, the pain intensity ranges between 3 to 6 on the numerical pain scale, but lesions at the level of the brainstem and thalamus report higher values [5]. The pain is often characterized as either constant “burning,” “freezing,” “aching,” or intermittent “shooting” [5]. Sensory abnormalities such as thermal (cold) or pinprick sensations are noted in approximately 90% of the patients [10].

Different hypotheses have been presented to explain the pathophysiological mechanisms that cause post-stroke pain. Central sensitization postulates that central nervous system (CNS) lesions result in neurochemical, anatomical, and inflammatory changes that trigger neuronal excitability leading to pain [11]. Another hypothesis describes hyperexcitability specifically in the spinothalamic tract, which evokes thermal and pinprick stimuli abnormalities [12]. Likewise, the thermosensory disinhibition theory suggests that the loss of normal inhibition of pain from the lesion causes an imbalance in the signaling pathway between the medial and lateral spinothalamic tract [5]. The complex interconnections in the brain allow for different theories to arise. The dynamic reverberation theory explains that post-stroke pain arises due to improper oscillatory patterns in the loop running between the thalamus and cortex [13].

Current treatments for post-stroke pain management

The first-line treatment for post-stroke pain includes tricyclic antidepressants, serotonin and norepinephrine reuptake inhibitors, or calcium channel ligands [14-18]. Second-line treatment includes antiepileptics such as carbamazepine or lamotrigine [19,20]. Third-line therapeutic agents include ketamine, intravenous lidocaine infusions, or steroids [21-24]. Clinicians have begun shifting toward the utility of medications that antagonize the N-methyl D-aspartate (NMDA) receptor for neuropathic pain, as their efficacy is equivocal to other treatments [25]. NMDA antagonists include amantadine, methadone, ketamine, and dextromethorphan [25]. Because of the potential for abuse, opioids are not used for long-term therapy to treat post-stroke neuropathic pain [26].

Studies have revealed that current treatment options specified in the guidelines of the international pain society are not as effective in improving neuropathic pain in post-stroke patients [27-30]. For example, neither carbamazepine nor levetiracetam was significantly effective in small, randomized trials [7]. Carbamazepine is an NMDA antagonist that works by blocking the effects of NMDA, resulting in neuroprotective effects [25]. Reported adverse effects include hepatotoxicity and psychomotor effects like sedation, lack of concentration, and ataxia [25]. Carbamazepine has been shown to statistically significantly decrease pain measured using a visual analog scale and has been shown to be as efficacious as pregabalin and venlafaxine, but neither was significantly effective in reducing post-stroke pain [25]. However, due to undesirable side effects such as ataxia, its use is undesirable in post-stroke patients [25].

Neurostimulation for post-stroke pain is an active research area [27-29]. Stimulating the motor cortex can modulate pain pathways that then activate inhibitory pathways between neutral structures and pathways [27]. Non-invasive stimulation, such as repetitive transcranial magnetic stimulation (rTMS) and transcranial direct current stimulation (tDCS), are fundamental for functional recovery after stroke [28,29]. However, the risks outweigh the benefits in most post-stroke patients [27-29].

Ischemic stroke enhances neuroinflammation by activating increased microglia, macrophages, macrophages, TNF alpha, and inflammatory cytokines [31]. TNF alpha plays a major role in the inflammatory response. Neural brain injuries, such as traumatic brain injury, spinal cord injury, and middle cerebral artery occlusion, signal microglia and macrophages to release TNF alpha [2]. Types of TNF alpha include soluble (sol) and transmembrane (tm) types, which can contribute to the complex inflammatory response seen during the stroke [32]. TNF alpha can cross the blood-brain barrier, further exacerbating the inflammatory response injured brain cells produce [31]. TNF alpha levels correlate with the volume associated with the brain infarct and sharply increase and persist immediately after the stroke [2]. Neuroinflammation activated by TNF alpha is commonly seen in the sequelae following strokes [32]. Etanercept, a TNF alpha inhibitor, has been shown to improve inflammation that leads to post-stroke pain [32]. Therefore, this literature review aims to assess and review the role of etanercept in the management of post-stroke pain.

## Review

Methods and results

In this review, databases including PubMed, Google Scholar, and Cochrane were searched. The search criteria included post-stroke pain and perispinal etanercept. A total of 12 articles were selected based on the search criteria and are summarized in the data extraction table shown in Table 1.

**Table 1:** Data extraction table TNF alpha: tumor necrosis factor alpha.

Reference	Purpose	Study type	Study description	Key findings
Clark (2022) [33]	To explore how perispinal etanercept can affect the mechanisms of long-COVID and post-stroke syndromes.	Review article	Analyze various randomized clinical trials that study perispinal etanercept’s effect on post-stroke pain and the role of TNF alpha in the development of post-stroke pain.	Excess TNF alpha production in the CSF is associated with post-stroke pain. Perispinal etanercept can enter the CSF and inhibit TNF alpha production to treat post-stroke pain.
Tobinick et al. (2012) [34]	To systematically evaluate the effect of perispinal etanercept on patients with neurological dysfunctions following a stroke or traumatic brain injury (TBI).	Observational study	A total of 629 patients were evaluated: 617 stroke patients and 12 TBI patients. The mean age of patients was 65.8 years. The mean time between treatment and stroke was 42 months.	Perispinal administration of etanercept significantly improved motor dysfunctions, spasticity, sensory dysfunctions, cognition, aphasia, and pain. Improvements were noted irrespective of the time before treatment.
Menter et al. (2019) [35]	To examine the various biologic agents that can be used to treat psoriasis.	Clinical guideline	Discuss mechanisms of action, benefits, and side effects of biological agents for psoriasis including etanercept.	Etanercept is a TNF-alpha inhibitor that is FDA-approved for the treatment of psoriasis, rheumatoid arthritis, and ankylosing spondylitis.
Ghezzi et al. (1991) [36]	To evaluate the mechanisms of increased production of TNF alpha and interleukin-1 (IL-1).	Experimental article	Discuss the mechanisms behind hypoxia-induced production of TNF alpha and IL-1, aggravating various conditions.	Hypoxia resulted in increased production of TNF alpha and IL-1.
Enbrel (2022) [37]	To outline the prescribing information of a brand type of etanercept.	Prescribing information pamphlet	Discuss the prescribing instructions for etanercept.	Subcutaneous etanercept’s dose ranges from 25 mg/0.5 mL to 50 mg/mL. Enbrel is FDA-approved for plaque psoriasis, rheumatoid arthritis (RA), psoriatic arthritis, ankylosing spondylitis (AS), and juvenile idiopathic arthritis.
Zhou et al. (2011) [38]	To evaluate the pharmacokinetics of etanercept.	Integrated analysis	In this integrated analysis, 53 healthy volunteers, 212 rheumatoid arthritis patients, and 346 ankylosing spondylitis patients were included.	Disease type, age, health status, and patient’s body weight did not affect the pharmacokinetics of etanercept.
Lovell et al. (2006) [39]	To evaluate the safety and efficacy of four-year treatment with etanercept in patients with juvenile rheumatoid arthritis (JRA).	Randomized control trial	A total of 69 patients enrolled in the study, 34 of whom received etanercept for four or more years. Safety was evaluated by using rates of serious adverse events and serious infections. Efficacy was evaluated by using the American College of Rheumatology (ACR) Pediatric 30 criteria for improvement and standard measures of disease activity.	Serious adverse events occurred at a rate of 0.13 per patient per year, and the rate of serious infections was 0.04 per patient per year. A total of 94% of patients treated for four or more years with etanercept yielded efficacious responses.
Yim et al. (2005) [40]	To evaluate the pharmacokinetics and efficacy of etanercept dosed at either 0.4 mg/kg twice weekly or 0.8 mg/kg once weekly.	Integrated analysis	A total of 69 pediatric JRA patients aged between four and 17 years were included in the analysis. A mixed-effect analysis and Monte Carlo clinical trial stimulation experiment were done.	The 0.8 mg/kg once weekly dosing was found to be equivalent to the 0.4 mg/kg twice weekly dosing of etanercept. This led to the FDA approving 0.8 mg/kg once weekly subcutaneous dosing for pediatric JRA patients.
Ralph et al. (2015) [41]	To evaluate evidence for TNF alpha inhibition by using etanercept in patients with neuropathic pain due to strokes or TBI.	Review article	Review randomized clinical trials evaluating perispinal etanercept on chronic stroke patients with neuropathic pain.	TNF alpha is involved in depression, migraines, and neuropathic pain. Perispinal etanercept has been found to improve neuropathic pain in chronic stroke patients and TBI patients with neurological dysfunctions.
Ralph et al. (2020) [42]	To identify the effectiveness of perispinal etanercept for patients with chronic post-stroke pain.	Randomized control trial	Participants in the study were assigned to either the control (saline) or the test group (perispinal etanercept). The main outcome assessed was the difference in pain levels between the two groups.	There was a statistically significant decrease in pain after the administration of perispinal etanercept; no change was noted in the control group. A total of 33% of those in the test group noted pain cessation after the first dose of perispinal etanercept.
Tobinick (2011) [43]	To report three patients with long-standing neurological deficits due to stroke, and their responses to perispinal etanercept.	Case series	Three patients who had strokes were treated with perispinal etanercept after failing rehabilitation and previous treatment.	All three patients had significant improvement after the perispinal etanercept. Improvement was noted in gait and hand function along with sensory deficits, speech, and cognition.
Tobinick (2020) [44]	To report a post-stroke patient’s response to the clock drawing test after receiving perispinal etanercept.	Case report	A 59-year-old man with chronic left hemiparesis due to a right middle cerebral artery stroke presented with post-stroke pain for 16 years, and was administered perispinal etanercept to assess response.	Within 10 minutes after perispinal etanercept administration, the reported chronic left-sided pain was gone. The patient’s Fatigue Assessment Score had decreased by seven points following perispinal etanercept administration. After 17 days of treatment, the patient noted the absence of pain and allodynia.

Proposed role of TNF alpha in post-stroke pain

TNF alpha is known to maintain cerebral homeostasis mainly but has been shown to increase in pathological states like infection or stroke [33]. The excess production of TNF alpha led to increased pain symptoms following these states, such as post-stroke pain [33,34]. Therefore, one possible mechanism to decrease post-stroke pain is decreasing TNF alpha [33]. Even though TNF alpha is too large to cross the blood-brain barrier, etanercept can inhibit TNF alpha when administered perispinally [33].

TNF alpha is a pro-inflammatory cytokine produced by the body as part of the normal immune response to pathogens and infections. It increases endothelial selectin (E-selectin) expression and induces the production of acute-phase proteins via the liver [35]. Levels of TNF alpha can be affected by several factors. For instance, a cerebral infarction leads to an inflammatory state that causes an increased production of TNF alpha by monocytes [36]. Also, TNF alpha increases to precipitate neurite outgrowth and cell differentiation by nerve growth factor [33]. TNF alpha also helps regulate the strength of normal synaptic transmission in neuronal activity [33].

Studies have shown that chronic inflammatory conditions, such as post-stroke syndrome, are due to TNF alpha chronically activating microglia with a damage-associated molecular patterns (DAMP) molecule, thereby producing excess amounts of TNF alpha in the cerebrospinal fluid (CSF) [33]. This positive feedback loop has also been implicated in ischemic stroke and traumatic brain injury [33]. The endotoxin tolerance, normally observed after the resolution of an inflammatory state, is observed systematically throughout the body but not in the brain [33]. The lack of cerebral endotoxin tolerance results in the positive feedback loop that chronically produces excess TNF alpha only in the CSF [33]. Therefore, a TNF alpha inhibitor such as etanercept can stop the positive feedback loop causing the excess TNF alpha production in post-stroke pain by being administered directly through the CSF [33].

Pharmacokinetics and pharmacodynamics of etanercept

Etanercept is a soluble dimer form of a 75 kilodalton protein (p75), which is one of the two specific receptor types that TNF alpha binds to on the cell surface [35]. Etanercept binds to the active trimeric form of TNF alpha to inhibit function [35]. This decreases the circulation of TNF alpha, thereby decreasing the level of serum cytokines, E-selectin, and matrix metalloproteinases [35]. Its actions include ligand function for both high and low-affinity immunoglobulin gamma Fc region receptors [35].

For most conditions, etanercept is given via subcutaneous injection [37]. The dosage varies depending on what condition it is used to treat and the age of the patient [37]. Solution dosage ranges from 25 milligrams/0.5 milliliter (mg/mL) to 50 mg/mL via prefilled syringes [38]. For adults with plaque psoriasis, rheumatoid arthritis (RA), or ankylosing spondylitis, 25 mg/0.5 mL syringes are prescribed twice weekly, and 50 mg/mL syringes are prescribed once weekly [35]. For pediatric patients aged two years to adolescence with juvenile idiopathic arthritis (JIA), subcutaneous solutions are also used and depend on weight [35]. Children weighing less than 65 kg can be given 0.8 milligrams/kilogram/dose once weekly with a maximum dose of 50 mg [39]. Children weighing more than 65 kg are given 50 mg once weekly [39]. The administration is done subcutaneously on the anterior side of the thigh and abdomen (avoiding the area around the navel or outer area of the upper arm) [39].

Etanercept’s onset of action is usually two to three weeks with maximum effect at three months [35]. It is absorbed slowly after subcutaneous injection, with a bioavailability of 60% [35]. Its half-life elimination in children aged four years and adolescents with JIA ranges from 31.2 to 104.8 hours while in adults (RA), it ranges from 72 to 132 hours [37,40].

Side effects of etanercept include reactivation of hepatitis B and latent tuberculosis, invasive fungal infections, and opportunistic infections [37]. Tuberculosis testing should therefore be done before initiating etanercept therapy [37]. Patients at risk for invasive fungal infections should be placed on empiric antifungal medications [37]. Cases of lymphoma, pancytopenia, new onset or exacerbation of psoriasis, and congestive heart failure have been shown in some patients on etanercept [37]. Live vaccinations should not be provided to patients on etanercept [37]. Etanercept use is contraindicated in patients placed on abatacept and cyclophosphamide due to the increased risk of serious adverse events [37].

Etanercept utilization in the post-stroke patient

Strokes are the second leading cause of functional disability worldwide [41]. The brain injury secondary to a stroke produces a persistent neuroinflammatory response in the brain, resulting in a spectrum of neurologic dysfunction known as post-stroke pain [41]. Excess production of TNF alpha in the CSF of stroke survivors has been implicated in post-stroke pain [33,41]. Several studies have shown statistically significant evidence that etanercept, a TNF alpha inhibitor, can reduce symptoms present in post-stroke syndrome by targeting the excess TNF alpha produced in the CSF [33,41,42].

Studies have shown that etanercept improves outcomes of post-stroke pain [41,42]. A double-blinded controlled clinical trial by Ralph et al. in 2020 showed that etanercept significantly improved chronic post-stroke pain [42]. A total of 30% of patients in the etanercept group had complete abatement of pain on the first treatment by etanercept, which was not seen in the control group [42]. The pain was measured using a visual analog scale [42]. After two perispinal etanercept treatments, the treatment group revealed a reduction in the worst measurable pain and average daily pain levels from baseline [42]. An observational study by Tobinick also revealed statistically significant improvements in outcomes of post-stroke patients, especially in motor dysfunction, spasticity, cognition, aphasia, and pain [41].

The outcomes were observed in patients irrespective of the time that passed before treatment with etanercept [34]. For instance, Tobinick reported a case series in which three consecutive patients with several post-stroke deficits were each administered perispinal etanercept 13, 35, and 36 months following their acute stroke, respectively [43]. Twenty-two to 26 days after the initial dose, a second dose was administered [43]. A statistically significant improvement was observed in all patients within 10 minutes of administration [43]. Additionally, Tobinick reported a 59-year-old post-stroke patient who, after receiving etanercept, showed immediate improvement in not only pain but also in hemispatial neglect [44]. These results suggest that etanercept shows clinical improvement and produces a rapid response in patients suffering from post-stroke deficits [34,43,44].

Studies have shown that etanercept provides relief in diseases other than chronic post-stroke pain. For instance, a large open-label trial led by Ralph et al. in 2015 reported that etanercept provided relief in not only post-stroke pain but also in cognitive function in dementia patients, neuropathic pain, and brain trauma [41]. When administered perispinally to the 600 patients included in the open-label trial, etanercept was found to be a safe, low-risk procedure that immediately improved their pain [41].

Limitations of the review process

This review included articles published only in English, due to the limited translational resources. This literature review focused on only TNF alpha since it is involved in the occurrence, development, and prognosis of stroke, although some studies do not support TNF alpha as a clear marker of stroke. Therefore, the effects of other selective TNF alpha inhibitors on post-stroke pain, and a side-by-side comparison with the effects of etanercept, were not included in this review.

Future research considerations

The use of TNF alpha as a main biomarker of stroke development or prognosis is a promising area of research to explore. Further research is required to understand the specific role and mechanism that TNF alpha plays in brain injury due to stroke and to minimize the side effects of anti-TNF alpha therapy. In addition, optimizing the anti-TNF alpha therapy regimen based on stroke severity and symptoms, patient characteristics, and timing of treatment should be further researched.

## Conclusions

Strokes are the second leading cause of death and functional disability worldwide. The brain injury resulting from stroke produces a persistent neuroinflammatory response in the brain, resulting in post-stroke pain. Excess production of TNF alpha in the CSF of stroke survivors has been implicated in post-stroke pain. Several studies have shown statistically significant evidence that etanercept, a TNF alpha inhibitor, can reduce symptoms present in post-stroke syndrome by targeting the excess TNF alpha produced in the CSF. Studies have also shown improvements in not only post-stroke pain but also in traumatic brain injury and dementia. Further research is required to explore the effects of TNF alpha on stroke prognosis and determine the optimal frequency and duration of etanercept treatment for post-stroke pain.
